# Survival and Results after Resection and Reconstruction with Megaprosthesis at the Hip in Octogenarians

**DOI:** 10.3390/jcm12247740

**Published:** 2023-12-17

**Authors:** Guido Scoccianti, Matteo Innocenti, Roberto Scanferla, Federico Scolari, Francesco Muratori, Andrea Ungar, Carlo Rostagno, Domenico Andrea Campanacci

**Affiliations:** 1 Department of Orthopaedic Oncology, Careggi University Hospital, 50134 Firenze, Italy; roberto.scanferla@unifi.it (R.S.); muratorif@aou-careggi.toscana.it (F.M.); domenicoandrea.campanacci@unifi.it (D.A.C.); 2 Orthopaedic Clinic, Careggi University Hospital, 50134 Firenze, Italy; matteo.innocenti@unifi.it; 3 Department of Health Sciences, University of Florence, 50121 Florence, Italy; federico.scolari@unifi.it; 4 Department of Internal and Geriatric Medicine, Careggi University Hospital, 50134 Firenze, Italy; andrea.ungar@unifi.it; 5 Department of Internal and Postoperative Medicine, Careggi University Hospital, 50134 Firenze, Italy; carlo.rostagno@unifi.it

**Keywords:** megaprostheses, very elder, geriatric surgery, bone tumors

## Abstract

Few data are available about results after procedures of resection and megaprosthesis at the hip in very elderly patients. The aim of our study was to ascertain survival and complications in patients aged 80 or older undergoing these major orthopedic procedures. A consecutive series of 27 procedures in 26 patients aged 80–93 years was evaluated. In total, 15 procedures were performed due to oncological diseases, 6 were performed following joint arthroplasty failures or periprosthetic fractures, and 6 were performed after trauma or trauma sequelae. Survival of the patients ranged from 0 to 122 months. Overall survival was 56% at 3 years, 24% at 5 years, and 16% at 8 years. An early postoperative death during the first 3 months occurred in five patients (18.5%). The only preoperative parameter negatively affecting survival was preoperative hemoglobin lower than 11 g/dL. Local complications were similar to reported rates in all-age patients’ series. In our experience, resection and megaprosthetic reconstruction can also be a valid choice in very elderly patients, with 56% of patients living more than two years from surgery and 24% more than five. Nevertheless, early postoperative deaths are frequent. A multidisciplinary evaluation of frailty of the patient must be accomplished, and patients and relatives must be informed about the risks of the procedure.

## 1. Introduction

During the past few decades, improved perioperative care facilities have made major surgical procedures feasible in elderly patients. This trend also occurred in orthopedic surgery, and the use of megaprostheses—either for oncological diseases or for failures after arthroplasty or trauma surgery—also progressively increased in elderly or very elderly (80 years or older) patients. Nonetheless, few data are available specifically concerning the results of resections and megaprosthesis reconstructions in this particular subset of the population. To the best of our knowledge, there are no prospective or retrospective studies addressing only this particular group of patients. Even if most published series included patients older than 80 and often even 90 years (until 97 years, as reported by Viste et al. [1]), it is very difficult to draw from these data any conclusion about the results of the procedures in the very elderly. Also, the few papers which addressed the issue of megaprosthetic reconstructions in elderly patients included in the analysis also patients between 65 and 79 years who present different frailty profiles in comparison with octogenarians [2,3]. Early functional recovery to avoid bed-ridden related complications and allow autonomy in daily-life activities is of utmost importance in elderly patients, but local and systemic complications remain a matter of concern when performing major orthopedic surgery in the very elderly.

The aim of our study was to answer the following questions:

- What is the survival rate in octogenarian patients after surgical procedures of resection and reconstruction with a megaprosthesis at the hip?
- What are the incidence and characteristics of complications after these procedures in particularly frail patients, as very elderly patients are?

## 2. Materials and Methods

A retrospective analysis of all patients treated at our institution with proximal or total femoral resection and reconstruction with a megaprosthesis from 2000 to 2020 was performed.

The eligibility criteria for this study were as follows:

- Age 80 years or older;
- Patients receiving a femoral resection (proximal or total femur) and reconstruction with a megaprosthesis at the hip;
- A minimum follow-up of 24 months unless death occurred before.

Both patients affected by oncological and non-oncological diseases were included, and a comparison analysis between the two groups was performed.

The Waldemar Link Megasystem-C megaprosthetic (Waldemar Link GmbH & Co., Hamburg, Germany) system was the implant used in all patients. Due to the age of the patients, a fixation with cement of the stem was performed in all patients. The rehabilitation schedule varied according to the specific case and comorbidities, with a protected full weight-bearing progressive gait restoration allowed as soon as the general conditions of the patient permitted.

Antibiotic prophylaxis was usually performed with preoperative intravenous vancomycin and tobramycin unless allergy or renal impairment caused a different choice. Antibiotic treatment was continued after surgery until drainage removal. In recent years, we have been using piperacillin–tazobactam instead of tobramycin.

Perioperative and follow-up data were collected from medical records, and when recent data or definitive data (death reported) were lacking, we performed a clinical evaluation when feasible or a phone interview (with the patient or with his/her relatives in case of death).

Preoperative data collected included disease determining surgical indication for resection (tumor, failure of previous arthroplasty, trauma sequelae); histotype (in oncological patients); age; anatomical site; type of megaprosthesis implanted; presence of heart disease determining chronic medical treatment (including ischemic, arrhythmic, and valvular or dilated cardiopathy) and/or previous cardiac surgery; presence of clinically significant comorbidities other than heart disease (including diabetes, renal failure, chronic obstructive pulmonary disease, cirrhosis of the liver); and preoperative levels of hemoglobin and total protein serum concentration. Postoperative data collected included the number of perioperative blood transfusions; the number and type of complications with their treatment and outcome; the level of gait recovery after surgery (no recovery, walking with a walker trolley or two crutches, walking with one crutch or cane, or walking without aids); and the length of survival.

The study was performed in accordance with the 1975 Declaration of Helsinki. The study was retrospective and approved by the local institutional Ethical Committee.

### Statistical Analysis

Statistical analysis was performed using software R version v 4.3.0 (https://www.r-project.org, accessed on 15 september 2023). Descriptive analysis was performed to report frequencies, rates, and a synthesis of qualitative variables. The non-parametric Kaplan–Meier estimator was used to assess the survival rate of the entire series. A comparison of the survival distribution among different groups of patients was performed using the long-rank test (Mantel–Cox test), considering *p*-values of less than 0.05 statistically significant with a 95% confidence interval, and comparing estimates of the hazard functions of different groups of patients (stratified using the above-mentioned parameters) in order to ascertain whether there were factors with a negative effect on prognosis (overall survival).

## 3. Results

From 2000 to 2020, we performed 27 resections (26 proximal femur resections; 1 total femur resection) and reconstructions with a megaprosthesis at the hip in 26 patients aged 80 or older. One patient received two subsequent proximal femur resections (both sides), with the second operation at 26 months from the first one.

All patients had died at the time of our final data gathering, except the last one of the series, who was alive at 28 months from surgery.

Age ranged from 80 to 93 years (mean 83.8; median 83). There were 14 female and 12 male patients (1 male patient received two procedures).

Fourteen patients (one undergoing 2 subsequent procedures due to bilateral disease) were affected by oncological diseases (8 metastatic lesions from carcinoma, including the 2 lesions of the patient bilaterally affected; 5 primary tumors; and 2 myelomas), three by late failures of joint arthroplasty procedures, three by periprosthetic fractures, and six by acute trauma or trauma sequelae not involving a pre-existing hip prosthesis implant.

**Figure 1:**
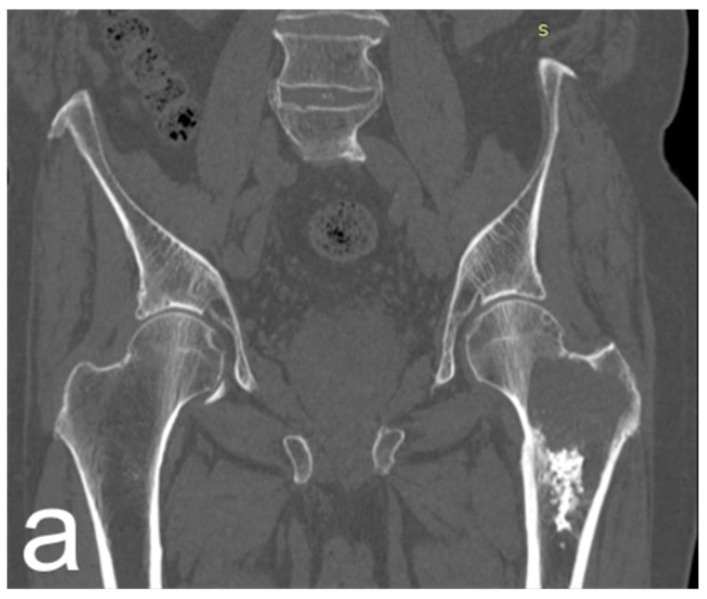

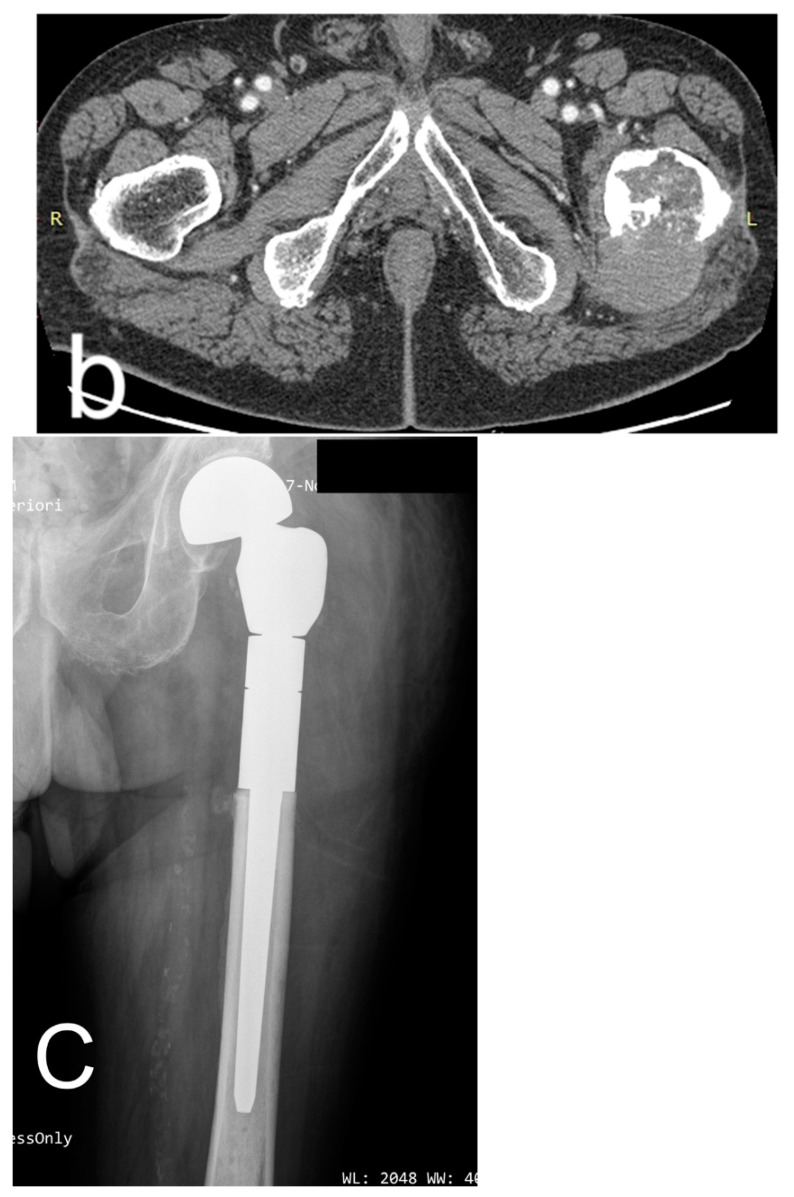
(**a**) Male, 84 years old, affected by dedifferentiated chondrosarcoma of the left proximal femur; CT coronal reconstruction of the proximal femur. (**b**) Axial CT scan shows extraosseous extension of the tumor. (**c**) Resection and reconstruction with a cemented proximal femur megaprosthesis were performed. The patient lived for 10 years (122 months) after surgery with no tumor recurrence and was able to walk with the aid of a cane. He died at 122 months due to a concomitant disease.

Characteristics of oncological patients are shown in Table 1. Among 15 procedures in tumor patients, one-third was performed for primary bone or soft tissue tumors, with chondrosarcoma representing the most frequent histotype, accounting for 80% of primary tumors and 100% of primary bone tumors (Figure 1).

For statistical evaluation, patients affected by metastatic disease and multiple myeloma were analyzed together.

In twenty-six procedures, proximal femur resection and megaprosthesis reconstruction were performed; one patient received a total femur prosthesis after total femur resection.

Data about comorbidities were available for 24 patients (25 procedures) and are shown in Table 2.

Due to the age of the population included, high blood pressure was a very common finding (75% of the patients) and therefore it was not considered among the parameters included in the analysis for prognostic significance.

Local complications included two deep infections, three prosthetic dislocations, one prosthetic breakage, one temporary peroneal nerve palsy, and one local tumor recurrence.

One of the two patients affected by deep infection (at 8 months from surgery) underwent a DAIR procedure (debridement, antibiotics, and implant retention); due to the early death of the patient, follow-up was too short to verify the long-term result of the procedure. In the second case, an early infection occurred with wound dehiscence; no additional surgical procedure could be performed because the patient died after a few days due to heart failure two months from surgery.

All three cases of dislocation were treated with closed reduction and a period of hip brace.

Mechanical failure of the prosthesis (one patient) was treated with revision surgery.

Three venous thromboses were detected during postoperative follow-up. On this issue, we want to highlight that we did not perform a systematic Doppler ultrasonography screening of our patient in the postoperative period. Therefore, the real incidence of deep thrombotic events could be higher, considering that such events can often be not clinically evident.

The average follow-up of the whole series was 41 months (median 39 months), considering the time from the first surgery for the patient receiving two subsequent procedures. The latest follow-up in the only alive patient was 28 months. The remaining 25 patients died at a time from surgery ranging from 0 to 122 months (average 42; median 40). One patient died in the perioperative period (day 5), and death occurred early (3 months or less) in four other patients; 18.5% was the cumulative rate of early death during the initial 3 months following surgery. 

According to Kaplan–Meier analysis, the whole series survival was 56% at 3 years, 24% at 5 years, and 16% at 8 years.

**Figure 2:**
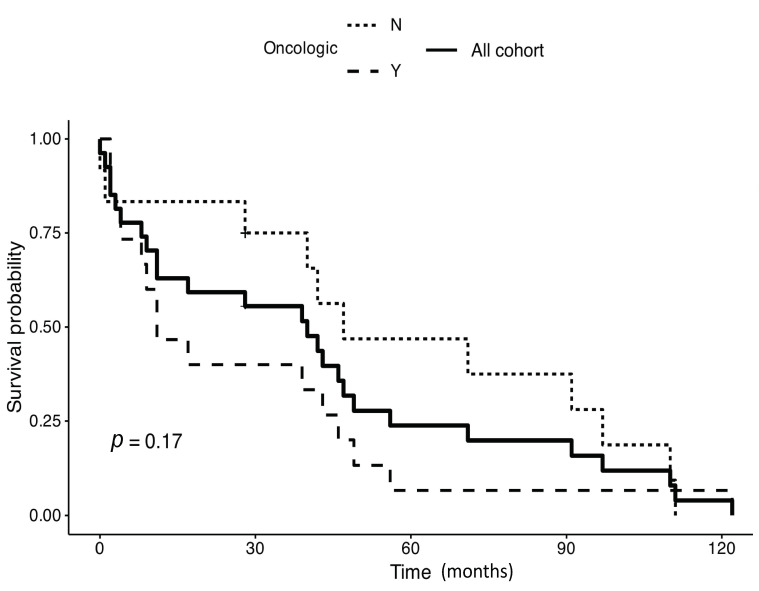
Survival of oncological patients (lower curve—Oncologic Y) versus non-oncological patients (upper curve—Oncologic N); log-rank test P 0.17. A wide gap between the two curves is apparent during the first years with survival afterward homogeneously decreasing in the long term due to the old age of patients at presentation. Also, the survival curve of the entire series is shown (mid curve—all cohort).

The whole series survival in oncological and non-oncological patients was not significantly different (*p* = 0.17; Figure 2). If we analyze the entire survival curves of these two groups, we find a survival of 40%, 7%, and 7% in oncological patients at 3, 5, and 8 years, respectively, and conversely, a survival of 75%, 47%, and 28% in non-oncological patients. Even if survival in the short term seems worse in oncological patients, a statistically significant difference in the comparison of the entire curves is not confirmed, and this may occur, in our opinion, because, as can be expected, the survival of patients who are so old at presentation will become homogeneously low in the very long term. In the group of oncological patients, we could not demonstrate a worse survival for patients who received surgery for a metastatic lesion (*p* = 0.18), but none of the patients affected by a secondary lesion survived for more than 4 years (49 months).

**Figure 3:**
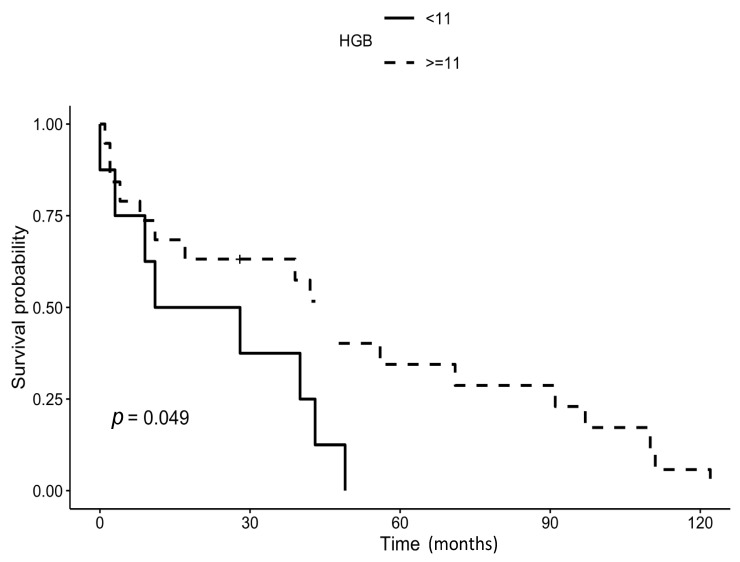
A comparison of survival of the patients according to preoperative hemoglobin value lower or equal/higher than 11 g/dL showed that the presence of a low value of preoperative hemoglobin negatively affected survival in our patients (*p* = 0.049).

The only preoperative parameter that negatively affected survival was preoperative hemoglobin value lower than 11 g/dL (*p* = 0.049; Figure 3); we did not find any significant relation between all the remaining assessed perioperative parameters and patient survival (Table 3). An analysis specifically addressing early mortality could not find any significant association between any of the evaluated parameters and mortality during the first three months after surgery (Fisher’s exact test; all parameters tested *p* > 0.55).

At follow-up, five patients were able to walk without aids, nine patients with one crutch or a cane (including the patient who received two subsequent megaprostheses, who could walk with one crutch after the first surgery, and with two crutches after the second surgery), and seven patients could walk with a walking trolley or two crutches. In one patient, no gait ability was present at the latest follow-up due to septic complication and early death afterward. In four patients, this parameter was not applicable due to early death (perioperative or before 3 months).

## 4. Discussion

Osteoarticular resection and reconstruction with megaprosthesis is a major surgical procedure. Results and complications of this technique were examined by several authors but, to the best of our knowledge, no published studies specifically addressed the particular group of very elderly patients.

Patients older than 80 years constitute a peculiar group of patients whose frailty deserves specific attention and a separate evaluation for results and complications. Decision-making in defining treatment in the elderly is always challenging, as the risk–benefit balance is particularly hard to evaluate, especially in patients affected by multiple comorbidities.

Our study has several limitations.

Firstly, it is a retrospective study, and this can introduce selection biases and incomplete data gathering. To reduce this limitation, we included in the study all the patients aged 80 or older in our series of femoral resections and megaprostheses of the hip over two decades of experience. It must also be mentioned that the rarity of the procedures in the very elderly and the need for a long follow-up to ascertain survival make it very difficult to accomplish a prospective study on such an issue.

Secondly, our study includes both oncological and non-oncological patients. Overall survival of the whole series can thus be misleading for one group or the other, but in the analysis, we also examined each group separately, and a comparison between the two groups was performed.

Thirdly, the number of patients included in the study is limited, and this makes it very difficult to draw significant conclusions. The authors are aware of this problem, and they want the readers to be aware of it, too. Nonetheless, due to the rarity of the indications of a similar procedure in the very elderly, it is quite hard to reach high numbers of patients, and the lack of data in the literature can make the report of even a small series interesting to try to focus attention and begin to shed light on a controversial and poorly investigated issue.

Considering all these limitations, the results of our study show that a high early mortality rate is to be expected in a population of very elderly patients undergoing resection surgery and megaprosthetic reconstruction. Nevertheless, long survival and efficient gait recovery are possible and do occur in a significant portion of patients. More than half of our patients (54%) lived more than three years after surgery, and almost a quarter of them (23%) lived more than five years after surgery.

In our experience, most patients could regain gait ability, with 14 out of 26 patients (54%) obtaining a valid restoration of walking independence (just one crutch or cane or no aids at all).

We evaluated several perioperative parameters, from blood parameters to the perioperative number of blood transfusions or the presence of comorbidities, in order to ascertain if any of them could affect the survival of the patients. In our series, only the level of preoperative hemoglobin showed a negative effect on survival. A relation between preoperative hemoglobin levels and postoperative survival in orthopedic surgery was already found by other authors, particularly concerning hip fractures in old patients [4,5,6,7]. Our data confirm this finding in the particular subset of elderly patients undergoing resection and megaprosthetic reconstruction at the hip. Severe thinness (body mass index <16.00) was found by other authors to be a risk factor for postoperative adverse events in elderly patients undergoing surgery for soft tissue sarcomas [8], but we could not include this parameter in our analysis due to the lack of this datum in the first patients of the series. The preoperative level of serum total proteins, which can be considered another parameter related to the health and nutrition status of the patient, did not show in our series a correlation with survival. Our failure to find factors affecting patient prognosis can be a consequence of the small number of patients included in the study, but it surely highlights the need for a comprehensive score, able to simultaneously include several parameters that can detect patient frailty.

To the best of our knowledge, such a tool specifically studied for elderly patients undergoing major orthopedic surgery is still lacking.

A parameter that has been widely validated for perioperative morbidity risk is the American Society of Anesthesiologists-Physical Status (ASA-PS) classification, which was found to be an efficient risk index in malignant bone and soft tissue tumors [9], but few papers can be found using specific post-operative morbidity scores in orthopedic surgery [10], and they normally use tools initially developed in different fields of surgery, such as the Estimation of Physiologic Ability and Surgical Stress [11,12] or the surgical risk calculator proposed by the American College of Surgeons [13]. Overall geriatric assessment scores have been applied by several authors to patients undergoing orthopedic surgery, but this was usually carried out in trauma surgery (hip fractures) or standard elective arthroplasty, with a lower age selection cut-off and with the target of evaluating the first-month or first-year morbidity/mortality results without data in the very long term. The Clinical Frailty Scale [14] was not found to be a predictive factor for postoperative complications in a series of 23 patients treated with megaprosthesis after an articular fracture at the lower limb at the age of 72.87 ± 12.33 years [15]. Moreover, reported results are not unanimous on the efficacy of the numerous single parameters or comprehensive tools assessed and on the identification of the best tools to be used [15,16,17,18,19,20,21,22].

Developing a specific comprehensive evaluation score to predict postoperative morbidity and survival in major orthopedic surgery could be a useful aid for surgeons dealing with high-risk patients, such as elderly patients, undergoing major orthopedic surgical procedures.

Nonetheless, even if dedicated scores can be developed, a case-specific comprehensive medical evaluation is obviously of utmost importance and cannot be replaced by any mathematical score. Therefore, specialized geriatric evaluation and perioperative care are strongly advised in elderly patients [10,23,24,25,26].

An interesting finding of our study is that, even though secondary bone tumors are by far more frequent than primary tumors in this subset of the population, primary bone tumors do occur also in old and very old patients. Five patients in our series were affected by primary tumors (four bone sarcomas; one soft tissue sarcoma). Choice of surgical treatment must always consider this possibility, and a preliminary biopsy must always be performed unless the patient is already known to be affected by multiple bone metastases.

As could be expected in a series of patients 80 years or older, chondrosarcoma was the most frequent histotype among primary tumors.

Both in oncological and non-oncological patients, reconstruction with a megaprosthesis allows an earlier mobilization and full weight-bearing in comparison with other complex reconstructive techniques. This is particularly important and useful in very elderly patients, but the rate of local complications is high. A review, including data from 49 studies (2721 patients) about megaprostheses in tumor surgery, reported a rate of reoperation for implant/surgery-related failures of 41%, excluding tumor recurrences [27].

Deep infection is one of the most devastating complications in megaprosthesis surgery. Its incidence continues to be high, with meta-analysis of multiple studies showing an average rate of 9–10% [27,28]. Meta-analysis studies including only proximal femur resections found an average rate of infection of 5.1 to 7.6% (range 0–33%) in this specific subset of megaprosthetic implants [29,30,31,32].

Two cases of deep infection occurred among our 27 procedures, with a 7.4% rate, which is similar to the average rate found in the series, which include all-age patients.

Another frequent complication in proximal femur megaprostheses is dislocation due to the extensive soft tissue removal and the difficulty of achieving a secure repair of the residual soft structures. In a meta-analysis addressing proximal femur resections average dislocation rate of proximal femur megaprostheses varied from 5.1 to 15.7%, with a value ranging from 0 to 40% in the analyzed series [29,30,31,32]. Such a wide range is likely to be due also to the different proportion in the reported series of patients undergoing hemi-arthroplasties—as most often performed in tumor surgery—and total hip arthroplasties, as prevalently used in non-oncologic surgery. If we look at the meta-analysis including only megaprostheses implanted for non-oncologic conditions, an average rate of dislocation from 10.2 to 15.7% was reported [30,31], in comparison with an average rate of 5.1 to 5.4 in meta-analysis addressing only oncologic patients [29,32]. In our study, concerning a mixed population of patients undergoing hemiarthroplasties and total hip arthroplasties, 26 proximal femur and 1 total femur megaprostheses were implanted, and three patients suffered a hip dislocation (11.1%).

On the basis of these results, local postoperative complication rates found in our population of very elderly patients seem comparable to the rates reported in the series, including all-age patients.

Conversely, a particularly high early mortality was found, with five patients (18.5%) passing away in the first 3 months after surgery, highlighting how threatening and frequent systemic complications can be in this subset of patients.

## 5. Conclusions

Resection and megaprosthesis reconstruction can be a valid choice even in very elderly patients, with more than half of the patients analyzed in this study living more than three years from surgery and about one-fourth living more than five years. Nevertheless, very early postoperative deaths are frequent (18.5% of patients died in the first 3 months). Multidisciplinary evaluation of the grade of frailty of the patients must be performed, and patients and relatives must be thoroughly informed about the risks of the procedure and the high rate of early fatal events. An intensive care unit must be available at the hospital to perform such a major surgery on elderly patients. Even if our study is based on a limited number of cases, we think that the results of our series can represent useful data about a scarcely investigated issue and, at the same time, a matter of growing interest for the orthopedic surgeon, either if devoted to orthopedic oncology or to joint arthroplasty or trauma. More extensive multicentric studies are needed to better investigate the outcome and define prognostic factors in this particular subset of patients. More than specific parameters singularly affecting survival, which are difficult to find and validate, particularly for a procedure that has rare indications, a comprehensive multiparametric preoperative evaluation score, based on the results of such studies, could be a useful tool for the orthopedic surgeon facing the difficult choice of whether to perform or not perform a resection procedure in the very elderly.

## Figures and Tables

**Table 1:** Histotypes of malignancies (n 15) affecting oncological patients.

	Histotype	Number
Primary Tumors	Chondrosarcoma	4
	Soft tissue pleomorphic sarcoma	1
	Kidney	4
Secondary Tumors	Breast	2
	Prostate	2
Hematological	Myeloma	2
Malignancies		

**Table 2:** Incidence of comorbidities (24 patients).

Comorbidities (38 Pts)	Number of Patients	Percentage
Heart disease	13	40.5
Diabetes	4	18.9
Chronic obstr pulm disease	2	10.8
Chronic renal failure	2	8.1
Cirrhosis of the liver	1	2.7

**Table 3:** Survival comparison between groups according to selected perioperative parameters (Mantel–Cox test). No statistically significant differences were detected between groups, except for preoperative hemoglobin level, with worse survival (*p* = 0.049) in patients presenting with preoperative hemoglobin values lower than 11 g/dL (see also Figure 3).

Perioperative Parameters and Survival	*p*
Preoperative hemoglobin: <11 vs. ≥11 g/dL	0.049
Oncological disease: yes vs. no	0.17
Heart disease: affected vs. not affected	0.25
Heart disease and other comorbidities: affected vs. not affected	0.40
Number of perioperative blood transfusions: ≤2 vs. >2	0.46
Preoperative serum total protein concentration: <6 vs. ≥6 g/dL	0.78

## Data Availability

The datasets used and/or analyzed during the current study are available from the corresponding author on reasonable request.
